# Supplementary material for: Transmission of ‘Candidatus Anaplasma camelii’ to mice and rabbits by camel-specific keds, Hippobosca camelina
Source: PLoS Negl Trop Dis. 2021 Aug 16;15(8):e0009671. doi: 10.1371/journal.pntd.0009671 (PMC8389426; doi:10.1371/journal.pntd.0009671)
Supplement: S1 Text — Table A in S1 Text: The feeding schedule of camel keds, Hippobosca camelina, on healthy Swiss white mice for pathogen transmission experiment. Detection of ‘Ca. Anaplasma camelii’ in experimental mice group was determined post-ked bites by PCR-HRM using genus-specific primers for 16S rRNA gene target. The data shows that 47.4% of mice in the test group (n = 9/19) have acquired Anaplasma infection following ked blood-feeding bites. The control mice group (n = 2) was not exposed to the biting flies. Table B in S1 Text: The feeding schedule of camel keds, H. camelina, on immunosuppressed mice (n = 60) for Anaplasma transmission to determine the effect of immunosuppression on mice infection. PCR-HRM analysis revealed Anaplasma infection rate of 6.9% in test mice (n = 4/58) two weeks post-ked bite exposure. Control mice were not exposed to fly bites. We recorded Anaplasma infection rate of 12.9% (n = 4/31) in mice after 60 days of follow-up screening, but all 27 mice that were sacrificed 140 days post-ked exposure were not infected with the Anaplasma sp. Table C in S1 Text: The feeding schedule of camel keds, H. camelina, on mice (n = 123) and rabbits (n = 6) for Anaplasma transmission study to determine vector competence of keds. PCR-HRM analysis targeting genus-specific 16S rRNA gene detected Anaplasma infection rates of 17.9% in test mice (n = 22/123) and in 25% of rabbits (n = 1/4) post-ked bites. Control mice (n = 8) and rabbits (n = 2) were not exposed to biting flies. Table D in S1 Text: The infection prevalence of ‘Ca. Anaplasma camelii’ in camels and camel keds in various seasons of the year; wet, late wet, and dry season. Table E in S1 Text: Presence of Anaplasma sp. in camel keds collected from camel herds in various geographical locations in Laisamis, northern Kenya. (DOCX) [file pntd.0009671.s001.docx]

**Table A in S1 Text: The feeding schedule of camel keds, *Hippobosca camelina,* on healthy Swiss white mice for pathogen transmission experiment.** Detection of ‘*Ca.* Anaplasma camelii’ in experimental mice group was determined post-ked bites by PCR-HRM using genus-specific primers for 16S rRNA gene target. The data shows that 47.4% of mice in the test group (*n* = 9/19) have acquired *Anaplasma* infection following ked blood-feeding bites. The control mice group (*n* = 2) was not exposed to the biting flies.

| **S/No.** | **Mouse ID** | **Total keds exposed**  (20 flies/mouse) | **Dates exposed**  (April 2018) | **Exposure frequency** | ***Anaplasma* infection status** |
| --- | --- | --- | --- | --- | --- |
| 1 | 1A (control 1) | Not applicable (N/A) | N/A | N/A | Negative |
| 2 | 1B (control 2) | N/A | N/A | N/A | Negative |
| 3 | 2A | 100 | 7^th^, 8^th^, 11^th^, 14^th^, 21^st^ | 5 | Negative |
| 4 | 2B | 100 | 7^th^, 8^th^, 11^th^, 14^th^, 21^st^ | 5 | *Anaplasma* sp. |
| 5 | 3A | 120 | 5^th^, 6^th^, 9^th^, 11^th^, 17^th^, 20^th^ | 6 | *Anaplasma* sp. |
| 6 | 3B | 120 | 5^th^, 6^th^, 9^th^, 11^th^, 17^th^, 20^th^ | 6 | Negative |
| 7 | 3C | 120 | 5^th^, 6^th^, 9^th^, 11^th^, 17^th^, 20^th^ | 6 | *Anaplasma* sp. |
| 8 | 4A | 100 | 10^th^, 12^th^, 17^th^, 18^th^, 21^st^ | 5 | Negative |
| 9 | 4B | 100 | 10^th^, 12^th^, 17^th^, 18^th^, 21^st^ | 5 | Negative |
| 10 | 5A | 100 | 5^th^, 6^th^, 9^th^, 12^th^, 15th | 5 | Negative |
| 11 | 5B | 100 | 5^th^, 6^th^, 9^th^, 12^th^, 15^th^ | 5 | Negative |
| 12 | 6A | 100 | 7^th^, 8^th^, 10^th^, 12^th^, 16^th^ | 5 | Negative |
| 13 | 6B | 100 | 7^th^, 8^th^, 10^th^, 12^th^, 16^th^ | 5 | Negative |
| 14 | 6C | 100 | 7^th^, 8^th^, 10^th^, 12^th^, 16^th^ | 5 | *Anaplasma* sp. |
| 15 | 7A | 60 | 11^th^, 13^th^, 16 | 3 | Negative |
| 16 | 7B | 60 | 11^th^, 13^th^, 16 | 3 | *Anaplasma* sp. |
| 17 | 8 | 40 | 11^th^, 15^th^ | 2 | *Anaplasma* sp. |
| 18 | 9A | 20 | 18^th^ | 1 | *Anaplasma* sp. |
| 19 | 9B | 20 | 18^th^ | 1 | Negative |
| 20 | 10A | 20 | 19^th^ | 1 | *Anaplasma* sp. |
| 21 | 10B | 20 | 19^th^ | 1 | *Anaplasma* sp. |

**Table B in S1 Text: The feeding schedule of camel keds, *H. camelina,* on immunosuppressed mice (*n* = 60) for *Anaplasma* transmission to determine the effect of immunosuppression on mice infection.** PCR-HRM analysis revealed *Anaplasma* infection rate of 6.9% in test mice (*n* = 4/58) two weeks post-ked bite exposure. Control mice were not exposed to fly bites. We recorded *Anaplasma* infection rate of 12.9% (*n* = 4/31) in mice after 60 days of follow-up screening, but all 27 mice that were sacrificed 140 days post-ked exposure were not infected with the *Anaplasma* sp.

| S/No. | Mouse ID | Total keds exposed  (20 flies/mouse) | Date(s) exposed | Exposure frequency | *Anaplasma* infection status |
| --- | --- | --- | --- | --- | --- |
| 1 | Control 1 | Not applicable (N/A) | N/A | N/A | Negative |
| 2 | Control 2 | N/A | N/A | N/A | Negative |
| 3 | 1A | 60 | 28.06.18, 3.07.18, 4.07.18 | 3 | Negative |
| 4 | 1B | 60 | 28.06.18, 3.07.18, 4.07.18 | 3 | Negative |
| 5 | 1C | 60 | 28.06.18, 3.07.18, 4.07.18 | 3 | Negative |
| 6 | 1D | 60 | 28.06.18, 3.07.18, 4.07.18 | 3 | Negative |
| 7 | 1E | 60 | 28.06.18, 3.07.18, 4.07.18 | 3 | Negative |
| 8 | 1F | 60 | 28.06.18, 3.07.18, 4.07.18 | 3 | *Anaplasma* sp. |
| 9 | 1G | 60 | 28.06.18, 3.07.18, 4.07.18 | 3 | Negative |
| 10 | 1H | 60 | 28.06.18, 3.07.18, 4.07.18 | 3 | Negative |
| 11 | 1I | 60 | 28.06.18, 3.07.18, 4.07.18 | 3 | Negative |
| 12 | 1J | 60 | 28.06.18, 3.07.18, 4.07.18 | 3 | Negative |
| 13 | 1K | 60 | 28.06.18, 3.07.18, 4.07.18 | 3 | Negative |
| 14 | 1L | 60 | 28.06.18, 3.07.18, 4.07.18 | 3 | Negative |
| 14 | 2A | 20 | 29.06.2018 | 1 | Negative |
| 16 | 2B | 20 | 29.06.2018 | 1 | Negative |
| 17 | 2C | 20 | 29.06.2018 | 1 | *Anaplasma* sp. |
| 18 | 2D | 20 | 29.06.2018 | 1 | Negative |
| 19 | 2E | 20 | 29.06.2018 | 1 | Negative |
| 20 | 3A | 40 | 27.06.18 & 4.07.18 | 2 | Negative |
| 21 | 3B | 40 | 27.06.18 & 4.07.18 | 2 | Negative |
| 22 | 3C | 40 | 27.06.18 & 4.07.18 | 2 | Negative |
| 23 | 3D | 40 | 27.06.18 & 4.07.18 | 2 | *Anaplasma* sp. |
| 24 | 4A | 20 | 29.06.2018 | 1 | Negative |
| 25 | 4B | 20 | 29.06.2018 | 1 | *Anaplasma* sp. |
| 26 | 4C | 20 | 29.06.2018 | 1 | Negative |
| 27 | 4D | 20 | 29.06.2018 | 1 | Negative |
| 28 | 4E | 20 | 29.06.2018 | 1 | Negative |
| 29 | 4F | 20 | 29.06.2018 | 1 | Negative |
| 30 | 4G | 20 | 29.06.2018 | 1 | Negative |
| 31 | 4H | 20 | 29.06.2018 | 1 | Negative |
| 32 | 4I | 20 | 29.06.2018 | 1 | Negative |
| 33 | 5A | 20 | 26.06.2018 | 1 | Negative |
| 34 | 5B | 20 | 26.06.2018 | 1 | Negative |
| 35 | 6A | 20 | 26.06.2018 | 1 | Negative |
| 36 | 6B | 20 | 26.06.2018 | 1 | Negative |
| 37 | 6C | 20 | 26.06.2018 | 1 | Negative |
| 38 | 6D | 20 | 26.06.2018 | 1 | Negative |
| 39 | 6E | 20 | 26.06.2018 | 1 | Negative |
| 40 | 6F | 20 | 26.06.2018 | 1 | Negative |
| 41 | 6G | 20 | 26.06.2018 | 1 | Negative |
| 42 | 6H | 20 | 26.06.2018 | 1 | Negative |
| 43 | 6I | 20 | 26.06.2018 | 1 | Negative |
| 44 | 7A | 20 | 3.07.2018 | 1 | Negative |
| 45 | 7B | 20 | 3.07.2018 | 1 | Negative |
| 46 | 7C | 20 | 3.07.2018 | 1 | Negative |
| 47 | 7D | 20 | 3.07.2018 | 1 | Negative |
| 48 | 7E | 20 | 3.07.2018 | 1 | Negative |
| 49 | 7F | 20 | 3.07.2018 | 1 | Negative |
| 50 | 7G | 20 | 3.07.2018 | 1 | Negative |
| 51 | 7H | 20 | 3.07.18 | 1 | Negative |
| 52 | 8A | 60 | 4.07.18, 8.07.18, 9.07.18 | 3 | Negative |
| 53 | 8B | 60 | 4.07.18, 8.07.18, 9.07.18 | 3 | Negative |
| 54 | 8C | 60 | 4.07.18, 8.07.18, 9.07.18 | 3 | Negative |
| 55 | 8D | 60 | 4.07.18, 8.07.18, 9.07.18 | 3 | Negative |
| 56 | 9A | 20 | 4.07.18, 8.07.18, 9.07.18 | 1 | Negative |
| 57 | 9B | 20 | 4.07.18, 8.07.18, 9.07.18 | 1 | Negative |
| 58 | 9C | 20 | 4.07.18, 8.07.18, 9.07.18 | 1 | Negative |
| 59 | 9D | 20 | 4.07.18, 8.07.18, 9.07.18 | 1 | Negative |
| 60 | 9E |  | 4.07.18, 8.07.18, 9.07.18 | 1 | Negative |

**Table C in S1 Text: The feeding schedule of camel keds, *H. camelina*, on mice (*n* = 123) and rabbits (*n* = 6) for *Anaplasma* transmission study to determine vector competence of keds.** PCR-HRM analysis targeting genus-specific 16S rRNA gene detected *Anaplasma* infection rates of 17.9% in test mice (*n* = 22/123) and in 25% of rabbits (*n* = 1/4) post-ked bites. Control mice (*n* = 8) and rabbits (*n* = 2) were not exposed to biting flies.

| **S/No.** | **Mouse ID** | **Total keds exposed**  (20 flies/mouse) | **Dates exposed**  (July-August 2019) | **Exposure frequency** | ***Anaplasma* infection status** |
| --- | --- | --- | --- | --- | --- |
| 1. **Mice experiments:** | | | | | |
| 1 | 1A (Control) | 0 | Not exposed to biting flies including keds | 0 | Negative |
| 2 | 1B (Control) | 0 |  | 0 | Negative |
| 3 | 1C (Control) | 0 |  | 0 | Negative |
| 4 | 1D (Control) | 0 |  | 0 | Negative |
| 5 | 1E (Control) | 0 |  | 0 | Negative |
| 6 | 1F (Control) | 0 |  | 0 | Negative |
| 7 | 1G (Control) | 0 |  | 0 | Negative |
| 8 | 1F (Control) | 0 |  | 0 | Negative |
| 9 | 2A | 80 | 7^th^ July, 15^th^ July, 16^th^ July, 18^th^ July | 4 | Negative |
| 10 | 2B | 80 |  | 4 | *Anaplasma* sp*.* |
| 11 | 2C | 80 |  | 4 | *Anaplasma* sp*.* |
| 12 | 2D | 80 |  | 4 | *Anaplasma* sp*.* |
| 13 | 2E | 80 |  | 4 | *Anaplasma* sp*.* |
| 14 | 2F | 80 |  | 4 | *Anaplasma* sp. |
| 15 | 2G | 80 |  | 4 | *Anaplasma* sp. |
| 16 | 2H | 80 |  | 4 | Negative |
| 17 | 2I | 80 |  | 4 | *Anaplasma* sp. |
| 18 | 2J | 80 |  | 4 | Negative |
| 19 | 3A | 140 | 8^th^ July, 10^th^ July, 15^th^ July, 16^th^ July, 24^th^ July, 2^nd^ Aug, 3^rd^ Aug | 7 | *Anaplasma* sp. |
| 20 | 3B | 140 |  | 7 | Negative |
| 21 | 3C | 140 |  | 7 | Negative |
| 22 | 3D | 140 |  | 7 | Negative |
| 23 | 3E | 140 |  | 7 | Negative |
| 24 | 3F | 140 |  | 7 | Negative |
| 25 | 3G | 140 |  | 7 | Negative |
| 26 | 3H | 140 |  | 7 | Negative |
| 27 | 3I | 140 |  | 7 | Negative |
| 28 | 3J | 140 |  | 7 | Negative |
| 29 | 3K | 140 |  | 7 | Negative |
| 30 | 3L | 140 |  | 7 | Negative |
| 31 | 3M | 140 |  | 7 | Negative |
| 32 | 3N | 140 |  | 7 | Negative |
| 33 | 3O | 140 |  | 7 | Negative |
| 34 | 3P | 140 |  | 7 | Negative |
| 35 | 4A | 120 | 6^th^ July, 11^th^ July, 13^th^ July, 14^th^ July, 17^th^ July, 23^rd^ July | 6 | *Anaplasma* sp. |
| 36 | 4B | 120 |  | 6 | Negative |
| 37 | 4C | 120 |  | 6 | *Anaplasma* sp. |
| 38 | 4D | 120 |  | 6 | *Anaplasma* sp. |
| 39 | 4E | 120 |  | 6 | *Anaplasma* sp. |
| 40 | 4F | 120 |  | 6 | Negative |
| 41 | 4G | 120 |  | 6 | Negative |
| 42 | 4H | 120 |  | 6 | *Anaplasma* sp. |
| 43 | 4I | 120 |  | 6 | Negative |
| 44 | 4J | 120 |  | 6 | *Anaplasma* sp. |
| 45 | 5A | 80 | 23^rd^ July, 29^th^ July, 30^th^ July, 2^nd^ Aug | 4 | *Anaplasma* sp. |
| 46 | 5B | 80 |  | 4 | *Anaplasma* sp. |
| 47 | 5C | 80 |  | 4 | Negative |
| 48 | 5D | 80 |  | 4 | Negative |
| 49 | 5E | 80 |  | 4 | Negative |
| 50 | 5F | 80 |  | 4 | Negative |
| 51 | 5G | 80 |  | 4 | Negative |
| 52 | 5H | 80 |  | 4 | Negative |
| 53 | 6A | 80 | 8^th^ July, 28^th^ July, 29^th^ July, 30^th^ July | 4 | Negative |
| 54 | 6B | 80 |  | 4 | Negative |
| 55 | 6C | 80 |  | 4 | Negative |
| 56 | 6D | 80 |  | 4 | *Anaplasma* sp. |
| 57 | 6E | 80 |  | 4 | Negative |
| 58 | 6F | 80 |  | 4 | Negative |
| 59 | 6G | 80 |  | 4 | Negative |
| 60 | 6H | 80 |  | 4 | Negative |
| 61 | 6I | 80 |  | 4 | *Anaplasma* sp. |
| 62 | 7A | 80 | 23^rd^ July, 29^th^ July, 30^th^ July, 2^nd^ Aug | 4 | Negative |
| 63 | 7B | 80 |  | 4 | Negative |
| 64 | 7C | 80 |  | 4 | Negative |
| 65 | 7D | 80 |  | 4 | Negative |
| 66 | 7E | 80 |  | 4 | Negative |
| 67 | 7F | 80 |  | 4 | Negative |
| 68 | 7G | 80 |  | 4 | Negative |
| 69 | 7H | 80 |  | 4 | Negative |
| 70 | 8A | 180 | 15^th^ July, 17^th^ July, 18^th^ July, 19^th^ July, 20^th^ July, 21^st^ July, 28^th^ July, 29^th^ July, 30^th^ July | 9 | Negative |
| 71 | 8B | 180 |  | 9 | Negative |
| 72 | 8C | 180 |  | 9 | Negative |
| 73 | 8D | 180 |  | 9 | Negative |
| 74 | 8E | 180 |  | 9 | Negative |
| 75 | 8F | 180 |  | 9 | Negative |
| 76 | 8G | 180 |  | 9 | Negative |
| 77 | 8H | 180 |  | 9 | Negative |
| 78 | 8I | 180 |  | 9 | Negative |
| 79 | 8J | 180 |  | 9 | Negative |
| 80 | 8K | 180 |  | 9 | Negative |
| 81 | 8L | 180 |  | 9 | Negative |
| 82 | 8M | 180 |  | 9 | Negative |
| 83 | 8N | 180 |  | 9 | Negative |
| 84 | 8O | 180 |  | 9 | Negative |
| 85 | 8P | 180 |  | 9 | Negative |
| 86 | 9A | 200 | 7^th^ July, 8^th^ July, 15^th^ July, 18^th^ July, 19^th^ July, 20^th^ July, 21^st^ July, 25^th^ July, 29^th^ July, 30^th^ July | 10 | Negative |
| 87 | 9B | 200 |  | 10 | Negative |
| 88 | 9C | 200 |  | 10 | Negative |
| 89 | 9D | 200 |  | 10 | Negative |
| 90 | 9E | 200 |  | 10 | Negative |
| 91 | 9F | 200 |  | 10 | Negative |
| 92 | 9G | 200 |  | 10 | Negative |
| 93 | 9H | 200 |  | 10 | Negative |
| 94 | 9I | 200 |  | 10 | Negative |
| 95 | 9J | 200 |  | 10 | Negative |
| 96 | 9K | 200 |  | 10 | Negative |
| 97 | 9L | 200 |  | 10 | Negative |
| 98 | 9M | 200 |  | 10 | Negative |
| 99 | 9N | 200 |  | 10 | Negative |
| 100 | 9O | 200 |  | 10 | Negative |
| 101 | 9P | 200 |  | 10 | Negative |
| 102 | 9Q | 200 |  | 10 | Negative |
| 103 | 9R | 200 |  | 10 | Negative |
| 104 | 10A | 80 | 25^th^ July, 28^th^ July, 29^th^ July, 30^th^ July | 4 | *Anaplasma* sp. |
| 105 | 10B | 80 |  | 4 | Negative |
| 106 | 10C | 80 |  | 4 | *Anaplasma* sp. |
| 107 | 10D | 80 |  | 4 | Negative |
| 108 | 10E | 80 |  | 4 | Negative |
| 109 | 10F | 80 |  | 4 | Negative |
| 110 | 10G | 80 |  | 4 | Negative |
| 111 | 10H | 80 |  | 4 | Negative |
| 112 | 10I | 80 |  | 4 | Negative |
| 113 | 10J | 80 |  | 4 | Negative |
| 114 | 10K | 80 |  | 4 | Negative |
| 115 | 10L | 80 |  | 4 | Negative |
| 116 | 10M | 80 |  | 4 | Negative |
| 117 | 10N | 80 |  | 4 | Negative |
| 118 | 100 | 80 |  | 4 | Negative |
| 119 | 10P | 80 |  | 4 | Negative |
| 120 | 10Q | 80 |  | 4 | Negative |
| 121 | 10R | 80 |  | 4 | *Anaplasma* sp. |
| 122 | 10S | 80 |  | 4 | Negative |
| 123 | 10T | 80 |  | 4 | *Anaplasma* sp. |
| 1. **Rabbit experiments:** | | | | | |
| 1 | R1 | 120 | 10^th^ July, 13^th^ July, 16^th^ July, 20^th^ July, 22^nd^ July, 26^th^ July | 6 | Negative |
| 2 | R2 | 140 | 13^th^ July, 14^th^ July, 15^th^ July, 16^th^ July, 20^th^ July, 22^nd^ July, 26^th^ July | 7 | *Anaplasma* sp*.* |
| 3 | R3 | 140 | 13th July, 14th July, 15th July, 16th July, 20th July, 22nd July, 26th July | 7 | Negative |
| 4 | R4 | 140 | 13th July, 14th July, 15th July, 16th July, 20th July, 22nd July, 26th July | 7 | Negative |
| 5 | Jane (Control) | 0 | Not exposed to biting flies including keds | 0 | Negative |
| 6 | Jasmin (Control) | 0 |  | 0 | Negative |

The mice were divided into 10 groups; group one were the control mice that were not exposed to ked bites, whereas group 2 – 10 were the test mice which were exposed to ked bites at varying frequencies on different dates. R1 – R4 were the experimental rabbits exposed to the ked bites, whereas rabbits named Jane and Jasmin belonged to the control group.

**Table D in S1 Text: The infection prevalence of ‘*Ca*. Anaplasma camelii’ in camels and camel keds in various seasons of the year; wet, late wet, and dry season.**

| **Sampling site** | **Date** | **Season** | **Host** | **Prevalence of *Anaplasma* sp.** |
| --- | --- | --- | --- | --- |
| Koya  (N 01° 23' 11";  E 37° 57' 11.7") | September 2017 | Dry | Camels | 70.3% (175/249) |
|  |  |  | *H. camelina* | 9.9% (19/192) |
| Laisamis town  (N 01° 35' 16.1";  E 037° 48' 26.2") | June-July 2018 | Wet | Camels | 63.9% (179/280) |
|  |  |  | *H. camelina* | 20.8% (20/96) |
| Silapani  (N 01° 39’ 25.7”;  E 037° 49’ 00.6”) | July-August 2019 | Late wet | Camels | 77.9% (348/447) |
|  |  |  | *H. camelina* | 28.9% (22/76) |

**Table E in S1 Text: Presence of *Anaplasma* sp. in camel keds collected from camel herds in various geographical locations in Laisamis, northern Kenya.**

| **Site** | **GPS coordinate** | **Year of sampling** | **Prevalence of *Anaplasma* in keds** |
| --- | --- | --- | --- |
| Silapani | N01° 39’ 25.7”  E037° 49’ 00.6” | 2019 | 14.85% (15/101) |
| Silapani | N01° 39’ 25.7”  E037° 49’ 00.6” | 2019 | 16.13% (5/31) |
| Silapani | N01° 39’ 25.7”  E037° 49’ 00.6” | 2019 | 16% (12/75) |
| Silapani | N01° 39’ 25.7”  E037° 49’ 00.6” | 2019 | 21.74% (5/23) |
| Sakardala -polytechnic | N01° 37’ 26.2”  E037° 48’ 31.6’’ | 2019 | 18.75% (6/32) |
| Nkwanja e Ndege |  | 2019 | 17.86% (10/56) |
| Kargi | N02° 31’ 0”  E037° 34’ 0” | 2019 | 8.7% (6/69) |
| Sirata | N01° 38’ 02.5”  E037° 48’ 22.7” | 2019 | 15% (3/20)  9.68% (3/31) |
| Sirata | N01° 38’ 02.5”  E037° 48’ 22.7 | 2018 | 33.33% (4/12) |
| Nkwanja e Ndege |  | 2018 | 19.35% (6/31) |
| Manyatta Secondary | N01° 34’ 48.9”  E037° 47’ 38.8’’ | 2018 | 32.14% (9/28) |
| Soweto | N01° 35’ 42.8”  E037° 48’ 40.4’’ | 2018 | 34.38% (11/32) |
| Tirgamo | N01° 35’ 39.8”  E037° 54’ 11.6’’ | 2018 | 25% (3/12) |
| Nkang e Lawai |  | 2018 | 25% (3/12) |
