# Supplementary material for: Transmission of ‘Candidatus Anaplasma camelii’ to mice and rabbits by camel-specific keds, Hippobosca camelina
Source: PLoS Negl Trop Dis. 2021 Aug 16;15(8):e0009671. doi: 10.1371/journal.pntd.0009671 (PMC8389426; doi:10.1371/journal.pntd.0009671)
Supplement: S1 Fig — One sequence from the test mouse (3A_M_C) and seven sequences, 8E - 35D, from naturally infected camels were 100% identical (MUSCLE v3.8) and BLAST of GenBank showed highest identity of 100% to ‘Ca. Anaplasma camelii’. (DOCX) [file pntd.0009671.s002.docx]

3A_M__C -------ATTCGTGCCAGCAGCCGCGGTAATACGGAGGGGGCAAGCGTTGTTCGGAATTA

8_E -----------------------------ATACGGAGGGGGCAAGCGTTGTTCGGAATTA

2_A -----------------------------------AGGGGGCAAGCGTTGTTCGGAATTA

43_B --------TCCGTGCCAGCAGCCGCGGTAATACGGAGGGGGCAAGCGTTGTTCGGAATTA

34_B CGGCAAACTCCGTGCCAGCAGCCGCGGTAATACGGAGGGGGCAAGCGTTGTTCGGAATTA

45_B CGGCAAACTCCGTGCCAGCAGCCGCGGTAATACGGAGGGGGCAAGCGTTGTTCGGAATTA

26_D CGGCAAACTCCGTGCCAGCAGCCGCGGTAATACGGAGGGGGCAAGCGTTGTTCGGAATTA

35_D CGGCAAACTCCGTGCCAGCAGCCGCGGTAATACGGAGGGGGCAAGCGTTGTTCGGAATTA

*************************

3A_M__C TTGGGCGTAAAGGGCATGTAGGCGGTTCGGTAAGTTAAAGGTGAAATGCCAGGGCTTAAC

8_E TTGGGCGTAAAGGGCATGTAGGCGGTTCGGTAAGTTAAAGGTGAAATGCCAGGGCTTAAC

2_A TTGGGCGTAAAGGGCATGTAGGCGGTTCGGTAAGTTAAAGGTGAAATGCCAGGGCTTAAC

43_B TTGGGCGTAAAGGGCATGTAGGCGGTTCGGTAAGTTAAAGGTGAAATGCCAGGGCTTAAC

34_B TTGGGCGTAAAGGGCATGTAGGCGGTTCGGTAAGTTAAAGGTGAAATGCCAGGGCTTAAC

45_B TTGGGCGTAAAGGGCATGTAGGCGGTTCGGTAAGTTAAAGGTGAAATGCCAGGGCTTAAC

26_D TTGGGCGTAAAGGGCATGTAGGCGGTTCGGTAAGTTAAAGGTGAAATGCCAGGGCTTAAC

35_D TTGGGCGTAAAGGGCATGTAGGCGGTTCGGTAAGTTAAAGGTGAAATGCCAGGGCTTAAC

************************************************************

3A_M__C CCTGGAGCTGCTTTTAATACTGCCAGACTCGAGTCCGGGAGAGGATAGCGGAATTCCTAG

8_E CCTGGAGCTGCTTTTAATACTGCCAGACTCGAGTCCGGGAGAGGATAGCGGAATTCCTAG

2_A CCTGGAGCTGCTTTTAATACTGCCAGACTCGAGTCCGGGAGAGGATAGCGGAATTCCTAG

43_B CCTGGAGCTGCTTTTAATACTGCCAGACTCGAGTCCGGGAGAGGATAGCGGAATTCCTAG

34_B CCTGGAGCTGCTTTTAATACTGCCAGACTCGAGTCCGGGAGAGGATAGCGGAATTCCTAG

45_B CCTGGAGCTGCTTTTAATACTGCCAGACTCGAGTCCGGGAGAGGATAGCGGAATTCCTAG

26_D CCTGGAGCTGCTTTTAATACTGCCAGACTCGAGTCCGGGAGAGGATAGCGGAATTCCTAG

35_D CCTGGAGCTGCTTTTAATACTGCCAGACTCGAGTCCGGGAGAGGATAGCGGAATTCCTAG

************************************************************

3A_M__C TGTAGAGGTGAAATTCGTAGATATTAGGAGGAACACCAGTGGCGAAGGCGGCTATCTGGT

8_E TGTAGAGGTGAAATTCGTAGATATTAGGAGGAACACCAGTGGCGAAGGCGGCTATCTGGT

2_A TGTAGAGGTGAAATTCGTAGATATTAGGAGGAACACCAGTGGCGAAGGCGGCTATCTGGT

43_B TGTAGAGGTGAAATTCGTAGATATTAGGAGGAACACCAGTGGCGAAGGCGGCTATCTGGT

34_B TGTAGAGGTGAAATTCGTAGATATTAGGAGGAACACCAGTGGCGAAGGCGGCTATCTGGT

45_B TGTAGAGGTGAAATTCGTAGATATTAGGAGGAACACCAGTGGCGAAGGCGGCTATCTGGT

26_D TGTAGAGGTGAAATTCGTAGATATTAGGAGGAACACCAGTGGCGAAGGCGGCTATCTGGT

35_D TGTAGAGGTGAAATTCGTAGATATTAGGAGGAACACCAGTGGCGAAGGCGGCTATCTGGT

************************************************************

3A_M__C CCGGTACTGACGCTGAGGTGCGAAAGCGTGGGGAGCAAACAGGATTAGATACCCTGGTAG

8_E CCGGTACTGACGCTGAGGTGCGAAAGCGTGGGGAGCAAACAGGATTAGATACCCTGGTAG

2_A CCGGTACTGACGCTGAGGTGCGAAAGCGTGGGGAGCAAACAGGATTAGATACCCTGGTAG

43_B CCGGTACTGACGCTGAGGTGCGAAAGCGTGGGGAGCAAACAGGATTAGATACCCTGGTAG

34_B CCGGTACTGACGCTGAGGTGCGAAAGCGTGGGGAGCAAACAGGATTAGATACCCTGGTAG

45_B CCGGTACTGACGCTGAGGTGCGAAAGCGTGGGGAGCAAACAGGATTAGATACCCTGGTAG

26_D CCGGTACTGACGCTGAGGTGCGAAAGCGTGGGGAGCAAACAGGATTAGATACCCTGGTAG

35_D CCGGTACTGACGCTGAGGTGCGAAAGCGTGGGGAGCAAACAGGATTAGATACCCTGGTAG

************************************************************

3A_M__C TCCACGCTGTAAACGATGAGTGCTGAATGTGGGGACGTTTTGTCTCTGTGTTGTAGCTAA

8_E TCCACGCTGTAAACGATGAGTGCTGAATGTGGGGACGTTTTGTCTCTGTGTTGTAGCTAA

2_A TCCACGCTGTAAACGATGAGTGCTGAATGTGGGGACGTTTTGTCTCTGTGTTGTAGCTAA

43_B TCCACGCTGTAAACGATGAGTGCTGAATGTGGGGACGTTTTGTCTCTGTGTTGTAGCTAA

34_B TCCACGCTGTAAACGATGAGTGCTGAATGTGGGGACGTTTTGTCTCTGTGTTGTAGCTAA

45_B TCCACGCTGTAAACGATGAGTGCTGAATGTGGGGACGTTTTGTCTCTGTGTTGTAGCTAA

26_D TCCACGCTGTAAACGATGAGTGCTGAATGTGGGGACGTTTTGTCTCTGTGTTGTAGCTAA

35_D TCCACGCTGTAAACGATGAGTGCTGAATGTGGGGACGTTTTGTCTCTGTGTTGTAGCTAA

************************************************************

3A_M__C CGCGTTAAGCACTCCGCCTGGGGACTACGGTCGCAAGACTAAAACTCAAAGGAATTGACG

8_E CGCGTTAAGCACTCCGCCTGGGGACTACGGTCGCAAGACTAAAACTCAAAGGAATTGACG

2_A CGCGTTAAGCACTCCGCCTGGGGACTACGGTCGCAAGACTAAAACTCAAAGGAATTGACG

43_B CGCGTTAAGCACTCCGCCTGGGGACTACGGTCGCAAGACTAAAACTCAAAGGAATTGACG

34_B CGCGTTAAGCACTCCGCCTGGGGACTACGGTCGCAAGACTAAAACTCAAAGGAATTGACG

45_B CGCGTTAAGCACTCCGCCTGGGGACTACGGTCGCAAGACTAAAACTCAAAGGAATTGACG

26_D CGCGTTAAGCACTCCGCCTGGGGACTACGGTCGCAAGACTAAAACTCAAAGGAATTGACG

35_D CGCGTTAAGCACTCCGCCTGGGGACTACGGTCGCAAGACTAAAACTCAAAGGAATTGACG

************************************************************

3A_M__C GGGACCCGCACAAGCGGTGGAGCATGTGGTTTAATTCGATGCAACGCGAAGAACCTTACC

8_E GGGACCCGCACAAGCGGTGGAGCATGTGGTTTAATTCGATGCAACGCGAAGAACCTTACC

2_A GGGACCCGCACAAGCGGTGGAGCATGTGGTTTAATTCGATGCAACGCGAAGAACCTTACC

43_B GGGACCCGCACAAGCGGTGGAGCATGTGGTTTAATTCGATGCAACGCGAAGAACCTTACC

34_B GGGACCCGCACAAGCGGTGGAGCATGTGGTTTAATTCGATGCAACGCGAAGAACCTTACC

45_B GGGACCCGCACAAGCGGTGGAGCATGTGGTTTAATTCGATGCAACGCGAAGAACCTTACC

26_D GGGACCCGCACAAGCGGTGGAGCATGTGGTTTAATTCGATGCAACGCGAAGAACCTTACC

35_D GGGACCCGCACAAGCGGTGGAGCATGTGGTTTAATTCGATGCAACGCGAAGAACCTTACC

************************************************************

3A_M__C ACTTCTTGACATGGAGATTAGATCCTTCTTAACAGAAGGGCGCAGTTCGGCTGGATCTCG

8_E ACTTCTTGACATGGAGATTAGATCCTTCTTAACAGAAGGGCGCAGTTCGGCTGGATCTCG

2_A ACTTCTTGACATGGAGATTAGATCCTTCTTAACAGAAGGGCGCAGTTCGGCTGGATCTCG

43_B ACTTCTTGACATGGAGATTAGATCCTTCTTAACAGAAGGGCGCAGTTCGGCTGGATCTCG

34_B ACTTCTTGACATGGAGATTAGATCCTTCTTAACAGAAGGGCGCAGTTCGGCTGGATCTCG

45_B ACTTCTTGACATGGAGATTAGATCCTTCTTAACAGAAGGGCGCAGTTCGGCTGGATCTCG

26_D ACTTCTTGACATGGAGATTAGATCCTTCTTAACAGAAGGGCGCAGTTCGGCTGGATCTCG

35_D ACTTCTTGACATGGAGATTAGATCCTTCTTAACAGAAGGGCGCAGTTCGGCTGGATCTCG

************************************************************

3A_M__C CACAGGTGCTGCATGGCTGTCGTCAGCTCGTGTCGTGAGATGTTGGGTTAAGTCCCGCAA

8_E CACAGGTGCTGCATGGCTGTCGTCAGCTCGTGTCGTGAGATGTTGGGTTAAGTCCCGCAA

2_A CACAGGTGCTGCATGGCTGTCGTCAGCTCGTGTCGTGAGATGTTGGGTTAAGTCCCGCAA

43_B CACAGGTGCTGCATGGCTGTCGTCAGCTCGTGTCGTGAGATGTTGGGTTAAGTCCCGCAA

34_B CACAGGTGCTGCATGGCTGTCGTCAGCTCGTGTCGTGAGATGTTGGGTTAAGTCCCGCAA

45_B CACAGGTGCTGCATGGCTGTCGTCAGCTCGTGTCGTGAGATGTTGGGTTAAGTCCCGCAA

26_D CACAGGTGCTGCATGGCTGTCGTCAGCTCGTGTCGTGAGATGTTGGGTTAAGTCCCGCAA

35_D CACAGGTGCTGCATGGCTGTCGTCAGCTCGTGTCGTGAGATGTTGGGTTAAGTCCCGCAA

************************************************************

3A_M__C CGAGCGTAACCCTCATCCTTAGTTGCCAGCGGGTTAAGCCGGGCACTTTAAGGAGACTGC

8_E CGAGCGTAACCCTCATCCTTAGTTGCCAGCGGGTTAAGCCGGGCACTTTAAGGAGACTGC

2_A CGAGCGTAACCCTCATCCTTAGTTGCCAGCGGGTTAAGCCGGGCACTTTAAGGAGACTGC

43_B CGAGCGTAACCCTCATCCTTAGTTGCCAGCGGGTTAAGCCGGGCACTTTAAGGAGACTGC

34_B CGAGCGTAACCCTCATCCTTAGTTGCCAGCGGGTTAAGCCGGGCACTTTAAGGAGACTGC

45_B CGAGCGTAACCCTCATCCTTAGTTGCCAGCGGGTTAAGCCGGGCACTTTAAGGAGACTGC

26_D CGAGCGTAACCCTCATCCTTAGTTGCCAGCGGGTTAAGCCGGGCACTTTAAGGAGACTGC

35_D CGAGCGTAACCCTCATCCTTAGTTGCCAGCGGGTTAAGCCGGGCACTTTAAGGAGACTGC

************************************************************

3A_M__C CAGTGGTAAACTGGAGGAAGGTGGGGATGATGTCAAGTCAGCACGGCCCTTATGGGGTGG

8_E CAGTGGTAAACTGGAGGAAGGTGGGGATGATGTCAAGTCAGCACGGCCCTTATGGGGTGG

2_A CAGTGGTAAACTGGAGGAAGGTGGGGATGATGTCAAGTCAGCACGGCCCTTATGGGGTGG

43_B CAGTGGTAAACTGGAGGAAGGTGGGGATGATGTCAAGTCAGCACGGCCCTTATGGGGTGG

34_B CAGTGGTAAACTGGAGGAAGGTGGGGATGATGTCAAGTCAGCACGGCCCTTATGGGGTGG

45_B CAGTGGTAAACTGGAGGAAGGTGGGGATGATGTCAAGTCAGCACGGCCCTTATGGGGTGG

26_D CAGTGGTAAACTGGAGGAAGGTGGGGATGATGTCAAGTCAGCACGGCCCTTATGGGGTGG

35_D CAGTGGTAAACTGGAGGAAGGTGGGGATGATGTCAAGTCAGCACGGCCCTTATGGGGTGG

************************************************************

3A_M__C GCTACACACGTGCTACAATGGTGACTACAATAGGTTGCAATGTCGTAAGGCTGAGCTAAT

8_E GCTACACACGTGCTACAATGGTGACTACAATAGGTTGCAATGTCGTAAGGCTGAGCTAAT

2_A GCTACACACGTGCTACAATGGTGACTACAATAGGTTGCAATGTCGTAAGGCTGAGCTAAT

43_B GCTACACACGTGCTACAATGGTGACTACAATAGGTTGCAATGTCGTAAGGCTGAGCTAAT

34_B GCTACACACGTGCTACAATGGTGACTACAATAGGTTGCAATGTCGTAAGGCTGAGCTAAT

45_B GCTACACACGTGCTACAATGGTGACTACAATAGGTTGCAATGTCGTAAGGCTGAGCTAAT

26_D GCTACACACGTGCTACAATGGTGACTACAATAGGTTGCAATGTCGTAAGGCTGAGCTAAT

35_D GCTACACACGTGCTACAATGGTGACTACAATAGGTTGCAATGTCGTAAGGCTGAGCTAAT

************************************************************

3A_M__C CCGTAAAAGTCATCTCAGTTCGGATTGTCCTCTGCAACTCGAGGGCATGAAGTCGGAATC

8_E CCGTAAAAGTCATCTCAGTTCGGATTGTCCTCTGCAACTCGAGGGCATGAAGTCGGAATC

2_A CCGTAAAAGTCATCTCAGTTCGGATTGTCCTCTGCAACTCGAGGGCATGAAGTCGGAATC

43_B CCGTAAAAGTCATCTCAGTTCGGATTGTCCTCTGCAACTCGAGGGCATGAAGTCGGAATC

34_B CCGTAAAAGTCATCTCAGTTCGGATTGTCCTCTGCAACTCGAGGGCATGAAGTCGGAATC

45_B CCGTAAAAGTCATCTCAGTTCGGATTGTCCTCTGCAACTCGAGGGCATGAAGTCGGAATC

26_D CCGTAAAAGTCATCTCAGTTCGGATTGTCCTCTGCAACTCGAGGGCATGAAGTCGGAATC

35_D CCGTAAAAGTCATCTCAGTTCGGATTGTCCTCTGCAACTCGAGGGCATGAAGTCGGAATC

************************************************************

3A_M__C GCTAGTAATCGTGGATCAGCATGCCACGGTGAATACGTTCTCGGGTCTTGTACACACTGC

8_E GCTAGTAATCGTGGATCAGCATGCCACGGTGAATACGTTCTCGGGTCTTGTACACACTGC

2_A GCTAGTAATCGTGGATCAGCATGCCACGGTGAATACGTTCTCGGGTCTTGTACACACTGC

43_B GCTAGTAATCGTGGATCAGCATGCCACGGTGAATACGTTCTCGGGTCTTGTACACACTGC

34_B GCTAGTAATCGTGGATCAGCATGCCACGGTGAATACGTTCTCGGGTCTTGTACACACTGC

45_B GCTAGTAATCGTGGATCAGCATGCCACGGTGAATACGTTCTCGGGTCTTGTACACACTGC

26_D GCTAGTAATCGTGGATCAGCATGCCACGGTGAATACGTTCTCGGGTCTTGTACACACTGC

35_D GCTAGTAATCGTGGATCAGCATGCCACGGTGAATACGTTCTCGGGTCTTGTACACACTGC

************************************************************

3A_M__C CCGTCACGCCATGGGAATTGGCTTAACTCGAAGCTGGTGCGCCAACCGCAAGGAGGCAGC

8_E CCGTCACGCCATGGGAATTGGCTTAACTCGAAGCTGGTGCGCCAACCGCAAGGAGGCAGC

2_A CCGTCACGCCATGGGAATTGGCTTAACTCGAAGCTGGTGCGCCAACCGCAAGGAGGCAGC

43_B CCGTCACGCCATGGGAATTGGCTTAACTCGAAGCTGGTGCGCCAACCGCAAGGAGGCAGC

34_B CCGTCACGCCATGGGAATTGGCTTAACTCGAAGCTGGTGCGCCAACCGCAAGGAGGCAGC

45_B CCGTCACGCCATGGGAATTGGCTTAACTCGAAGCTGGTGCGCCAACCGCAAGGAGGCAGC

26_D CCGTCACGCCAT------------------------------------------------

35_D CCGTCACGCCATGGGAATTGGCTTAACTCGAAGCTGGTGCGCCAACCGCAAGGAGGCAGC

************

3A_M__C CATTTAAGGTTGGGTCAGTGACTAGGTGA

8_E CATTTAAGGTTGGGTCAGTGACTAGGTGA

2_A CATTTAAGGTTGGGTCAGTGACTAGGGTG

43_B CATTTAAGGTTGGGTCAGTGACTAGG---

34_B CATTTAAGGTTGGGTCAGTGACTAGGGTG

45_B CATTTAAGGTTGGGTCAGTGACTAGGGTG

26_D -----------------------------

35_D CATTTAAGGTTGGGTCAGTGACTAGGGTG

**S1 Fig: Multiple sequence alignment of eight ~1000-bp *Anaplasma* 16S rRNA genes.** One sequence from the test mouse (3A_M_C) and seven sequences, 8E - 35D, from naturally infected camels were 100% identical (MUSCLE v3.8) and BLAST of GenBank showed highest identity of 100% to ‘*Ca*. Anaplasma camelii’.
