# Supplementary material for: Transmission of ‘Candidatus Anaplasma camelii’ to mice and rabbits by camel-specific keds, Hippobosca camelina
Source: PLoS Negl Trop Dis. 2021 Aug 16;15(8):e0009671. doi: 10.1371/journal.pntd.0009671 (PMC8389426; doi:10.1371/journal.pntd.0009671)
Supplement: Attachment — Submitted filename: Response to Reviewers.docx [file pntd.0009671.s003.docx]

**Responses to reviewers’ comments:**

**Editors’ comments:**

Thanks for submitting your manuscript to PLoS Neglected Tropical Diseases. I have now received two independent reviews of your work, and as you will see, the reviewers raised some minor concerns about the research (mostly the manuscript format and content, although clarity is needed on experimental design in several cases). Despite this, these reviewers are optimistic about your work and the potential impact it will have on research studying vector biology and transmission dynamics of rickettsial species. Thus, I encourage you to revise your manuscript, accordingly, taking into account all of the concerns raised by both reviewers.

Please consider revising the title of your work to clearly indicate the scope/intent of the study. Also, please address the concerns regarding ked number and overall treatment of camels and why they specifically were investigated.

**Response**

We believe to have adequately addressed all the comments from the reviewers, which includes the concerns regarding ked numbers (please see lines 514 – 519; 551 – 554 of the revised manuscript) and overall treatment of camels (lines 126 – 132) and why they specifically were investigated (lines 71 – 73).

We also revisited the title of the study and decided to retain the original title (but with minor edits) as it reflects the major objective of our work on vector competence of keds in transmission of ‘*Candidatus* Anaplasma camelii’. We think that inclusion of details such as “Seasonal variation in the density of camel-specific keds” in the title could “dilute” the key message of the study. We would be happy to highlight the suggestions under “Keywords” as an alternative suggested by the Reviewer. Thus, our study title now reads: **“Transmission of ‘*Candidatus* Anaplasma camelii’ to mice and rabbits by camel-specific keds, *Hippobosca camelina*”.**

**Reviewer #1:**

Transmission of ‘*Candidatus* Anaplasma camelii’ to laboratory animals by camel-specific keds, *Hippobosca camelina* by Bargul et al.

**Reviewer’s summary**

The authors set out to determine the seasonal variation in the density of camel-specific keds, *Hippobosca camelina* and other livestock keds infesting livestock in the Laisamis area of Marsabit County, northern Kenya. In addition, they purposed to experimentally determine if *Hippobosca camelina* can transmit *Anaplasma* species to laboratory animals [Rabbits and mice]; datasets they discuss herein as important in extending the knowledge of the readership of PNTD towards the understanding of the role of *Hippobosca camelina* in mechanical transmission of *Anaplasma* species to *Camelus dromedarius* populations and other domestic animals. This manuscript presents novel datasets and I deem it helpful in the management of camel Anaplasmataceae infections. This is a good manuscript in its area which would further be improved by attending to the following minor changes;-

1. This manuscript presents data on the seasonal variation in the density of camel-specific keds, *Hippobosca camelina* and other livestock keds infesting livestock in the Laisamis area of Marsabit county, northern Kenya but this is not reflected in the current title or key words. I would suggest that the authors consider improving their manuscript title and key words to reflect this; e.g. “Seasonal variation in the density of camel-specific keds, Hippobosca camelina and demonstration of their experimental ability to transmission *Candidatus* Anaplasma camelii”.

**Response**

We thank the Reviewer for the comments on the study title, which we have taken time to think about. However, we wish to retain the original title with minor edits to highlight the key message on transmission of *Anaplasma* by keds. We would be happy to highlight the suggestions under “Keywords” as an alternative suggested by the Reviewer.

2. In this MS, where the term “small laboratory animals” is used should be replaced with “mice and rabbits

**Response**

Thank you for this suggestion.

We have now replaced “small laboratory animals” with “mice and rabbits” throughout the revised manuscript.

3. Rephrase and /or improve language use for sentences in lines; 14-16, 45-49, 155-158, 189-193, 270-276, 393-395,534-536, 638-640, 644-647, 647-650

**Response**

We thank the Reviewer for pointing these specific sentences lacking clarity in the manuscript. We have rephrased all these sentences to ensure clarity of information.

4. Line 202-203 and 233 …..with minor modifications; please state these minor modifications to allow for replication on the part of the reader

**Response**

The modifications are now clearly stated in the revised manuscript as follows;

The ability of camel keds, *H. camelina,* to transmit *Anaplasma* spp. to mice and rabbits was studied by following published protocols with minor modifications [23]. Camel keds were collected from camels and placed into well-ventilated cages covered with black netting. The keds were then released into larger cubical cages measuring 30 × 30 × 30 cm containing restrained mice for bloodmeal acquisition. Rabbits were shaved to reduce hair on their back to facilitate fly feeding. Freshly collected camel keds that had been kept in well-ventilated cages were then placed on the shaved back of rabbits to allow bloodmeal acquisition.

(please see lines 168-175).

5. Line 219;…..*Anaplasma* transmission; please specify the species of *Anaplasma*

**Response**

The *Anaplasma* species is now specified as ‘*Ca*. Anaplasma camelii’

6. Line 245; regularly….how regular?

**Response**

This is now clarified to mean that the mice and rabbits were screened for *Anaplasma* infection after two weeks post-ked bites.

(please see lines 210 – 211).

7. Lines 248… at intervals; please specify these intervals here

**Response**

This is now rephrased to mean the blood samples were collected from both control and test mice and rabbits after two weeks of ked exposure (please see lines 210 – 211).

8. Line 286; details are provided for DNA extraction from ked samples and not from camel, rabbit or mice blood samples. Please expand this section by providing details of how genomic/total DNA was extracted from the rest of the samples that were not keds

**Response**

Thank you, Reviewer.

This is now clarified as follows;

One hundred microliters (100 µL) of blood sample was pipetted into 1.5-mL Eppendorf tube and mixed with 20 µL proteinase K, and the total volume was adjusted to 220 µL with PBS at pH 7.4. Total genomic DNA was extracted from individual keds and blood samples from camels, mice, and rabbits using DNeasy Blood & Tissue Kit (Qiagen, Hilden, Germany) following the manufacturer’s instructions.

(please see lines 247 – 252).

9. Data analysis: Lines 348-349. Rephrase this sentence and provide details [e.g. packages/software extension, interpolation distance, etc] of how the maps were generated.

**Response**

This sentence is now rephrased as;

“A map of the sampling locations was created using geo-referenced data uploaded on the ArcMap extension of ArcGIS v 10.5 software by Esri.” Interpolation was not done.

(please see lines 292 – 293).

10. Lines 350-351: Provide details of packages used and the statistics that were computed; probably explaining your choice

**Response**

In our statistical analyses we used *glm* to determine how keds numbers on their hosts (counts) at different seasons and treatment conditions influenced the binary responses (disease outcomes; sick = 1 and healthy = 0). The default “*glm*() *function”* in R was used, and no specific package (e.g. lme4, nlme, or MASS, refer to packages for *glm’s* and associated methods; <https://cran.r-project.org/web/packages/cranly/vignettes/glms.html>) was employed in our computations. The choice of these statistical analyses is now well addressed in lines 306-310 of the revised manuscript.

11. Lines 422-443, 452-455, 460-464, 474-481: The details in these lines are part of methods and not results. Please consider deleting these details or if needed transferring them to the right sections of methodology

**Response**

We apologize for this mix-up of information.

These sections have now been edited in the manuscript to reflect the results and not methods.

12. Line 534; please specify the host whose blood smears were examined

**Response**

We have now rewritten this sentence in the revised manuscript to read;

“Field’s staining of thin blood smears prepared from camels, mice and rabbits, and examined to screen for presence of *Anaplasma* species revealed marginal occurrence of this pathogen in the host RBCs (Fig 4) similar to the well-studied *Anaplasma marginale.*”

(please see lines 460 – 462).

13. Line 553 or there about; please explain here how level of *Candidatus* Anaplasma camelii in experimentally infected laboratory animals compares with that of naturally infected camels and how this comparison gives merit to the possibility of natural transmission of *Candidatus* Anaplasma camelii by *Hippobosca camelina*.

**Response**

This is now explained in the Discussion section as follows;

We observed that the level of ‘*Ca.* Anaplasma camelii’ infection was higher in naturally infected camels than in experimentally infected mice and rabbits. Higher infection rates in camels could be attributed to infestation by other vectors such as ticks and *Stomoxys* that suck blood from camels and in the process spreading the *Anaplasma* pathogens. Also, keds could have better chances of mechanically transmitting *Anaplasma* pathogens among camels than in test mice and rabbits because their feeding is more likely to be interrupted by the camels’ response to painful fly bites, as opposed to restrained laboratory animals.

(please see lines 480 – 487).

14. Lines 554-559: to make the text presented in these lines valid, it would be good to explain why in experimentally testing immunity-based [immune status; suppression vs normal] factors as predictors of infection/transmission of *Candidatus* Anaplasma camelii, the authors did not keep exposure rate [number of keds] constant between groups!

**Response**

We were unable to keep ked exposure rates constant between different groups of mice because this depended on the available number of freshly collected ex-camel keds that were sometimes not sufficient to keep constant exposure rates with equal number of exposed flies in all three pathogen transmission experiments conducted in different seasons. For instance, unlike in the dry season with up to 100 keds/camel, wet season was characterized by relatively low numbers of keds infesting camels (seven keds/camel on average), thus we repeatedly exposed flies to blood-feed on the test mice. But importantly, the mice infections were achieved in all three repeat experiments. It was also impossible to determine *Anaplasma* contamination rates in live flies used in pathogen transmission experiments.

(please see lines 514 – 519; 551 - 555 of the revised manuscript for more clarification).

15. At Lines 590, 601 or there about, please discuss the likelihood of camel keds infesting or not infesting other livestock or even humans in the absence of camels; their preferred hosts.

**Response**

We have now included this information in the discussion section as follows;

Further, our studies show that *H. camelina* is camel-specific because it largely infested camels, but was hardly found on the other co-herded livestock species such as sheep, goats, cattle, donkeys, and dogs. Camels suffer the most burden from biting keds with greater infestations than all other co-herded livestock species, therefore making them most vulnerable to ked-borne diseases. (see lines 508-512)

Starved adult keds that departed from their vertebrate hosts will actively search for nearby vertebrate hosts, that include domestic and wild animals, as well as humans [45] and possibly spreading diseases to them. (see lines 519-521)

16. Lines 611-624: Please clarify why you would justify trap/target-based control methods for camel keds that spend > 10 hours attached and feeding on camels and not camel-based control methods e.g. insecticide application [Live bait technologies].

**Response**

We fully agree with the reviewer that insecticides could work better for controlling keds as the adult flies spend significant part of their lives on camels. However, keds detached from their vertebrate hosts are commonly found in the vegetation surrounding the grazing areas. We recommend use of traps for controlling these unattached keds that have the potential of transmiting zoonotic pathogens to humans and animals. Presently, there are no ked-specific insecticides or traps available for controlling keds.

(See lines 537-541).

**Reviewer #2:**

**Reviewer’s summary**

The paper is generally good with new findings; for example the presence of Anaplasma DNA in or on the camel keds is a novel finding. The work was done with adequate follow-up involving the local communities, who must have also learnt some thing new about the camel flies. The data is quite robust accompanied with detailed analysis. The presentation of Tables and Figures is of very high standards.

**Reviewer’s comments**

1. The authors have emphasized that camel flies are efficient fliers which is not the case. This statement is also contradicted by the same authors who have stated as follows in the same paper

“Both male and female keds are obligate blood feeders that stay with their camel hosts, unless disturbed” (Lines 86-88).

**Response**

We have now clarified this to mean that the camel flies can easily move from one camel host to the next one, in the same or between herds. Movement of flies occurs, for instance, when they are disturbed by the host in response to the painful blood-feeding bites inflicted by the flies (see lines 78-80 of the revised version).

2. If camel flies hardly leave hosts unless disturbed, then what is the likelihood of them being transmitters of pathogens in nature? (lines 183-185)

**Response**
Hematophagous adult keds spend most of their lives attached on the mammalian hosts. We observed that keds mainly infest the underbelly of the camels - mostly near the udder or male reproductive organs, and other parts including the neck, and hump (Kidambasi et al., 2020). We noticed that camels respond to the painful camel fly bites by hitting the fly-infested parts of its body using the legs, head, or the neck to ward off these nuisance biting keds. Consequently, the flies respond quickly by escaping or flying to safer locations on the camel’s body or may choose to move to other nearby hosts so as to avoid injury. Movements of keds is observed; (i) during milking of camels, (ii) in response to disturbance caused by the suckling calf, and (iii) in the process when camels sit down to rest.

We experienced challenges in collecting keds from camels in the daytime by handpicking as these flies quickly moved away. We discovered the solution towards efficient and fast collection of keds by hand was simply to conduct fly sampling at night under the cover of darkness when these flies seemed docile and with minimal movement on the camel’s body. However, keds became active even at night in presence of continuous artificial light source.

Camel flies, therefore, easily moved on or between the vertebrate hosts, and they may transmit hemopathogens that they carry during bloodmeal acquisition with high chances of frequent interrupted feeding due to painful bites they inflict on the hosts.

3. Mechanical transmission requires that the vector frequently jumps from host to host; if these flies infrequently depart from their camel host then it is unlikely that they play such a role in nature (line 613-614)

**Response**

We thank the reviewer for this constructive comment. We have deleted the words ‘infrequently depart’ as it could mislead the readers by implying that these flies are not disturbed by the host, hence there is no compelling reason for fly movement. Instead, keds move frequently on and between their vertebrate hosts as detailed above in Reviewer’s #2 comment #2. If the mouthparts of a ked are contaminated with hemopathogens, they could transmit the pathogens to the next host during interrupted blood feeding.

4. If *H. camelina* was found only on camels then what raw data was used to include other animal hosts in analysis as shown on Figure 3a? (Line 377-385).

**Response**

The key focus of our study was to characterize camel keds, *Hippobosca camelina,* and determine their role in disease transmission. Most farmers in our study region (Laisamis) practice co-herding of livestock species by co-grazing camels, goats, sheep, cattle, donkeys, and dogs. This motivated our survey on keds to establish whether camel keds could also infest the co-herded livestock species. Knowledge on the host range of *H. camelina* is important due to its implications on the spread of diseases, especially the zoonotic ones that affect humans. In this survey of keds infestations on livestock, we counted the host-attached keds using 8-key manual differential counter (Fisher Scientific, USA). Counting of keds was done during the day, and the same livestock herds were later traced for collection of keds at night with the help of trained field assistants selected from the local community. Keds samples were preserved for morphological and molecular identification. We barely found *H. camelina* on the co-herded livestock species, but they predominantly infested camels, suggesting host-specificity in *H. camelina*. The burden of infesting livestock keds seems to be heaviest on camels than in other livestock species studied.

(please see line 155 - 161).

5. Is the comparison on proportion of keds depicted on Figure 3a based on *H. camelina* or different ked species?

**Response**

The proportions of flies shown in Figure 3a was based on the total counts of keds on each individual host in a herd. Thus, we sampled all host-attached keds irrespective of its species, followed by sorting by sex, species, and host range.

Our preliminary findings based on morphological data shows that the ked samples collected from donkeys, cattle, goats, dogs, and sheep belong to different species in comparison to *Hippobosca camelina*.

The ongoing studies aim to; (i) characterize these livestock keds up to species level, and (ii) determine their role as disease vectors.

6. If *H. camelina* was found to be species-specific on the camel host, then why was it important to consider collections from other animal hosts such as donkeys, cattle, goats, dogs and sheep?

**Response**

Please refer to our response above to the **Reviewer’s comment #4** for relevant answers to this comment (as these two comments are linked).

In summary, since camels are always co-herded with other livestock species kept by the pastoralists in the study region, we undertook this experiment to study whether *H. camelina* infests these co-herded animals. However, only in a few instances was *H. camelina* identified in some sheep and goat herds co-herded with camels, with about 1 to 3 *H. camelina* flies collected in herds where the flies were present. *H. camelina* does not infest cattle, donkeys, and dogs.

7. Clarifications on reported information by the authors:

(a) Authors state that rates of contamination of keds with *Anaplasma* sp. varied in different geographical location, but this important statement is not supported with relevant data (line 552-553).

**Response**

Thank you for this observation. This data was provided in the manuscript under the supplementary information and is now cited accordingly as S5 Table in the corrected version of our manuscript (please see line 442).

(b) A statement suggests that the number of keds did not reduce during the wet season as the keds simply shifted from camel hosts to rest on the vegetation nearby (Line 595 to 598). This seems to contradict the findings that fly population numbers fluctuated with seasons.

**Response**

This is now clarified to mean that the number of camel-infesting keds sharply decreased in wet season, in contrast to the highest fly numbers of up to 100 keds/camel recorded during the dry season (see lines 514-519). We thus confirm these seasonal fluctuations in ked numbers on camels that impacted on our pathogen transmission experiments, particularly in the wet season which was characterized by limited of camel keds. In order to avoid confusion as rightly pointed out by the Reviewer, we have withdrawn earlier speculation attempting to explain the cause for reduction in ked numbers on camels during wet season; that these flies could possibly be departing from their camel host to search for new resting sites in the surrounding vegetation or near the animal grazing fields.

This statement is now deleted from the revised manuscript as it was speculative and we do not have any data to support this hypothesis.

(c) The method used to count the camel keds in a separate study to determine the seasonal fly populations is not specified (Line 191-193).

**Response**

This is now clearly stated in the revised manuscript as;

“Live keds on the camels and co-herded livestock species (cattle, goats, sheep, donkeys, and dogs) were counted using 8-key manual differential counter (Fisher Scientific, USA) during dry and wet seasons.” (Line 155-161)
